# The value of Lp(a) and TG/HDLC in peripheral blood to assess the stability of carotid plaque in patients with ischemic stroke

**DOI:** 10.1002/brb3.3355

**Published:** 2024-01-02

**Authors:** Yanan Tian, Lei Lu, Yazhao Zhang, Jianhui Wei

**Affiliations:** ^1^ Department of Neurology Harrison International Peace Hospital Hengshiu People's Republic of China; ^2^ Department of Neurosurgery Harrison International Peace Hospital Hengshiu People's Republic of China

**Keywords:** acute ischemic stroke, atherosclerosis of the carotid arteries, high‐density lipoprotein cholesterol, peripheral lipoprotein (a), triglycerides

## Abstract

**Objective:**

The objective of this study was to investigate the relationship between lipoprotein (a) (Lp(a)), triglyceride/high‐density lipoprotein cholesterol (TG/HDL‐C), and the stability of carotid atherosclerotic plaque in patients with acute ischemic stroke.

**Methods:**

A total of 142 patients with acute ischemic stroke were selected and divided into group A (59 cases of stable plaque formation) and group B (83 cases of unstable plaque formation) according to the characteristics of carotid artery plaque formation detected by carotid color Doppler ultrasound. The serum Lp(a), lipid metabolism indexes, peripheral blood routine indexes, and related serum inflammatory factors indexes were compared between the two groups. Receiver operating characteristic curve and multivariate logistic regression model were used to analyze the relationship between each index and the formation of carotid unstable plaque.

**Results:**

There were no significant differences in serum total cholesterol (TC), HDL‐C, and low‐density lipoprotein cholesterol (LDL‐C) between groups A and B (*p* > .05). The values of Lp(a), TG, and TG/HDL‐C in group B were higher than those in group A, and the differences were statistically significant (*p* < .05). There were no significant differences in serum TC, HDL‐C, and LDL‐C between groups A and B (*p* > .05). The values of Lp(a), TG, and TG/HDL‐C in group B were higher than those in group A, and the differences were statistically significant (*p* < .05). The values of HBA1C, Lp‐PLA2, CRP, CysC, Hcy, TNF‐α, neutrophils, and NLR in group B were higher than those in group A, and the differences were statistically significant (*p* < .05). There was no significant difference in FPG, systolic blood pressure, diastolic blood pressure, Hb, white blood cells, platelets, and lymphocytes between groups A and B (*p* > .05). The results of logistic regression model showed that the increase of Lp(a), TG/HDL‐C, HBA1C, Lp‐PLA2, CRP, CysC, Hcy, and NLR could increase the risk of carotid artery unstable plaque in patients with ischemic stroke (*p* < .05).

**Conclusion:**

Lp(a) and TG/HDL‐C have certain value in evaluating the stability of carotid atherosclerotic plaque in patients with acute ischemic stroke, and the increased levels of LP (a) and TG/HDL‐C will significantly increase the risk of carotid unstable plaque in patients.

## Graphical abstract

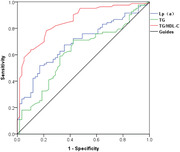

## 1 INTRODUCTION

Ischemic stroke is the major type of stroke occurring in the Chinese population, and ∼20% of ischemic stroke is closely associated with carotid atherosclerosis (Park et al., 2020; Paul & Candelario‐Jalil, 2021; Yang et al., 2021). Atherosclerosis plaques include stable and unstable plaques (Durham et al., 2018; Zhou et al., 2021). Related studies have shown that unstable plaques have a higher risk of ischemic stroke, and unanticipated detachment and rupture of unstable plaques are important factors in the development of ischemic stroke (Kopylov et al., 2017; Raggi, 2018). Studies have shown that peripheral lipoprotein(a) (Lp(a)) is an important influential factor in the development of cardiovascular disease, and monocytes and macrophages produce lipoprotein‐associated phospholipase A2 (Lp‐PLA2), and studies have also confirmed the close relationship between Lp‐PLA2 and atherosclerosis (Bittner et al., 2020; Najjar et al., 2018; Reyes‐Soffer & Westerterp, 2021). Lp‐PLA2‐mediated cytokines can promote matrix metalloproteinase in atherosclerosis plaques and protease expression, and matrix metalloproteinases degrade the fibrous cap and collagen matrix and other components of the plaque, leading to plaque rupture and bleeding, which leads to the development of ischemic stroke (J. Wang et al., 2021; F. Zhang et al., 2021). Low‐density lipoprotein (LDL) consists of large and small particles, and small‐particle LDL is more susceptible to oxidation and the formation of atherosclerosis plaques, and oxidized LDL is essential in the formation of vulnerable plaques (Ao & Chen, 2017). Several studies have shown that triglyceride/HDL cholesterol (TG/HDL‐C) can effectively reflect the size of LDL (Capón Álvarez et al., 2021; Wen et al., 2017). Therefore, this study investigated the relationship between Lp(a), TG/HDL‐C, and carotid atherosclerotic plaque stability in patients with ischemic stroke, aiming to provide valuable information for clinical diagnosis and treatment.

## 2 DATA AND METHODS

### 2.1 General information

One hundred and forty‐two eligible patients with acute ischemic stroke diagnosed from February 2019 to December 2021 in the Department of Neurology in Hengshui People's Hospital were selected as subjects. The patients were divided into two groups according to their carotid artery plaque formation characteristics as determined by carotid color Doppler ultrasonography: 59 patients in group A, all of whom had stable plaque formation in the carotid artery; 83 patients in group B, who had unstable type of carotid plaque formation.

Inclusion criteria were as follows: (1) the age range of patients was 19–79 years old; (2) the diagnostic criteria for patients with acute ischemic stroke referred to *the 2016 Guidelines for the diagnosis and treatment of acute ischemic stroke* (Chinese Society of Neurology & Chinese Stroke Society, 2018), and ischemic lesions were found by computed tomography and magnetic resonance imaging examination; (3) all patients were examined by carotid ultrasound, and the intima‐media thickness of the common carotid artery was found to be >1.5 mm with plaque formation; (4) patients were not treated with lipid‐lowering or other drugs affecting this study before admission; (5) the study protocol conformed to the regulations of the medical ethics committee, and informed consent was obtained from the study subjects or their families. Exclusion criteria were as follows: (1) intracranial tumor; (2) intracranial aneurysm, vascular malformation, hypertension, cerebral hemorrhage, and so on; (3) history of carotid artery surgery; (4) history of neck radiotherapy; and (5) serious infectious diseases.

### 2.2 Criteria for carotid ultrasound examination and plaque characteristics

Carotid ultrasonography was performed by a Philips IU22 color ultrasound machine with the patient lying flat and his/her neck fully exposed. The common carotid artery, external carotid artery, and internal carotid artery were examined from the top down, and the intima‐media thickness (IMT) of the carotid artery was measured to observe the intimal plaque situation in the lumen and recorded timely. According to *the Expert Consensus on Several Issues of Head and Neck Vascular Ultrasound (Carotid Artery Part)* (Dawkins et al., 2022), the criteria for the intima and plaque situation of the common carotid artery are as follows: IMT <1.0 mm is normal, 1.0 mm ≤ IMT < 1.5 mm is intimal thickening, and IMT ≥1.5 mm is plaque formation.

The main ultrasound echogenic features of unstable carotid plaque are hypoechoic, isoechoic, or inhomogeneous echogenicity; irregular plaque morphology; ulcer formation on or within the plaque surface; eccentricity index >2; and liquefied components inside the plaque.

### 2.3 Laboratory index inspection

Serum Lp(a), triglycerides (TG), total cholesterol (TC), high‐density lipoprotein cholesterol (HDL‐C), low‐density lipoprotein cholesterol (LDL‐C), fasting plasma glucose (FPG), glycated hemoglobin, type A1C (HBA1C), blood pressure index, lipoprotein‐associated phospholipase A2 (Lp‐PLA2), C‐reactive protein (CRP), photoprotein C (CysC), homocysteine (Hcy), tumor necrosis factor‐α (TNF‐α), hemoglobin (Hb), white blood cells (WBC), platelets (PLT), neutrophils (N), and lymphocytes (L) were compared between the two groups.

Serum Lp(a), TNF‐α, Lp‐PLA2, and CRP assay: 5 mL of fasting venous blood was collected from patients and centrifuged at 2000 r/min for 10 min, and the supernatant was stored at −80°C for use. Serum Lp(a) was measured by the immunoturbidimetric method, TNF‐α and Lp‐PLA2 were detected by ELISA, and CRP was detected by the immunoturbidimetric method.

Hcy and CysC assay: 5 mL of peripheral venous blood was taken from patients before treatment, placed in vacuum blood collection tubes containing ethylenediaminetetraacetic acid, and tested by automatic biochemical instrument.

Hb, WBC, PLT, N, and L assay: Patients were routinely examined after admission, and their Hb, WBC, PLT, N, and L data were recorded. Neutrophil lymphocyte ratio (NLR) was calculated by N/L.

TG, TC, HDL‐C, LDL‐C, FPG, and HBA1C test: Blood biochemical examination was performed after patients were admitted to the hospital, and the data of Hb, WBC, PLT, N, and L of patients were recorded.

Blood pressure index test: Blood pressure was measured using a blood pressure meter and recorded after the patients were admitted to the hospital.

### 2.4 Statistical methods

Data were processed with SPSS21.0, and the statistical description of the serum HBA1C, Lp‐PLA2, CRP, CysC, Hcy, TNF‐α, N, NLR, and other count data conforming to normal distribution collected in the study were completed by ($\bar{x}\pm s$). The hypothesis test for the comparison between the two groups of the above measurement data was done by an independent samples *t*‐test; count data (smoking, alcohol consumption, and gender) should be described by the number of cases (percentage), and the χ (Park et al., 2020) test was used for comparative analysis between groups; receiver operating characteristic curve (ROC) was used to diagnose the value of lipid metabolism indexes of carotid atherosclerotic plaque instability in patients with ischemic stroke; the analysis of risk factors for the presence of carotid unstable plaque in ischemic stroke patients was done by logistic regression model; and *p* < .05 indicated that the statistical differences were significant.

## 3 RESULTS

### 3.1 Comparison of general data between the two groups

There were no significant differences in age, body mass index (BMI), gender, smoking, and alcohol consumption between groups A and B (*p* > .05; Table 1).

**TABLE 1:** Comparison of general data of two groups of patients.

Group		Age(years)	BMI(kg/m^2^)	Gender(%)		Smoke(%)	Drink(%)
	*n*			Male	Female		
Group A	59	64.5 ± 7.0	24.01 ± 1.86	34 (57.63)	25 (42.37)	29 (49.15)	25 (42.37)
Group B	83	66.1 ± 8.2	24.26 ± 2.20	57 (96.61)	26 (44.07)	51 (86.44)	45 (76.27)
*t*/*X* ^2^		−1.216	−0.711	1.829		2.119	1.935
*p*		.226	.478	.176		.146	.164

Abbreviation: BMI, body mass index.

### 3.2 Comparison of lipid metabolism indexes between groups A and B

There were no significant differences in serum TC, HDL‐C, and LDL‐C between groups A and B (*p* > .05). The Lp(a), TG, and TG/HDL‐C values of group B were higher than those of group A, and the differences were statistically significant (*p* < .05; Table 2).

**TABLE 2:** Comparison of blood lipid metabolism indexes between groups A and B ($\bar{x}\pm s$).

Group	*n*	Lp(a)(mg/L)	TG(mmol/L)	TC(mmol/L)	HDL‐C(mmol/L)	LDL‐C(mmol/L)	TG/HDL‐C
Group A	59	279.0 ± 26.8	2.06 ± 0.35	5.29 ± 0.81	0.96 ± 0.11	3.22 ± 0.57	2.15 ± 0.11
Group B	83	296.5 ± 31.5	2.22 ± 0.41	5.50 ± 0.86	0.93 ± 0.10	3.40 ± 0.62	2.37 ± 0.14
*t*		−3.467	−2.432	−1.469	1.690	−1.762	−10.060
*p*		.001	.016	.144	.093	.080	.000

Abbreviations: HDL‐C, high‐density lipoprotein cholesterol; LDL‐C; low‐density lipoprotein cholesterol; Lp(a), lipoprotein(a); TC, total cholesterol; TG, triglycerides; TG/HDL‐C, triglyceride/high‐density lipoprotein cholesterol.

### 3.3 Value of lipid metabolism indexes in the diagnosis of carotid atherosclerotic plaque instability in patients with ischemic stroke

**FIGURE 1:**
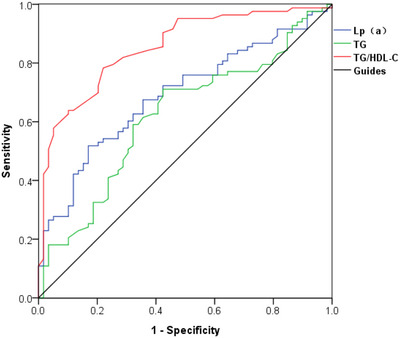
Receiver operating characteristic (ROC) curve of carotid atherosclerotic plaque in patients with ischemic stroke diagnosed by serum total cholesterol (TC), high‐density lipoprotein cholesterol (HDL‐C), and low‐density lipoprotein cholesterol (LDL‐C) as unstable plaque. LP(a), lipoprotein(a).

Serum Lp(a), TG, and TG/HDL‐C of patients in groups A and B were used to draw ROC curves. The results showed that the area AUC values of Lp(a), TG, and TG/HDL‐C for diagnosing carotid atherosclerotic plaque as unstable plaque in patients with ischemic stroke were 0.610, 0.619, and 0.854, respectively (Figure 1).

### 3.4 Comparison of blood routine, blood glucose, blood pressure, and inflammatory indexes between groups A and B

The values of HBA1C, Lp‐PLA2, CRP, CysC, Hcy, TNF‐α, N, and NLR in group B were higher than those in group A, and the differences were statistically significant (*p* < .05). There was no significant difference in FPG, systolic blood pressure, diastolic blood pressure, Hb, WBC, PLTs and L between groups A and B (*p* > .05; Table 3).

**TABLE 3:** Comparison of blood routine examination, blood glucose, blood pressure, and inflammatory indexes between groups A and B ($\bar{x}\pm s$).

Group	*n*	FPG(mmol/L)	HBA1C(%)	SBP(mmHg)	DBP(mmHg)	Lp‐PLA2(mg/L)	CRP(mg/L)	CysC(g/L)	Hcy(μmol/L)
Group A	59	5.90 ± 0.52	6.50 ± 0.68	134.8 ± 7.4	79.5 ± 5.7	224.1 ± 28.5	7.06 ± 1.68	1.60 ± 0.36	17.40 ± 2.56
Group B	83	6.05 ± 0.50	6.89 ± 0.74	137.0 ± 7.8	81.0 ± 6.2	241.7 ± 32.8	8.58 ± 2.01	1.85 ± 0.48	19.55 ± 2.84
*t*		−1.733	−3.200	−1.692	−1.469	−3.324	−4.747	−3.380	−4.629
*p*		.085	.002	.093	.144	.001	.000	.001	.000
Group	*n*	TNF‐α(ng/L)	Hb(g/L)	WBC(×10^9^/L)	PLT(×10^9^/L)	N(×10^9^/L)	L(×10^9^/L)	NLR	
Group A	59	8.15 ± 2.14	129.6 ± 5.6	6.80 ± 1.55	218.7 ± 18.6	5.14 ± 0.70	1.70 ± 0.38	3.02 ± 0.81	
Group B	83	9.82 ± 2.71	127.8 ± 6.2	7.14 ± 1.60	220.5 ± 20.5	5.56 ± 0.86	1.60 ± 0.41	3.48 ± 0.78	
*t*		−3.939	1.774	−1.264	−0.536	−3.092	1.476	−3.408	
*p*		.000	.078	.208	.593	.002	.142	.001	

Abbreviations: CRP, C‐reactive protein; CysC, photoprotein C; DBP, diastolic blood pressure; FPG, fasting plasma glucose; Hb, hemoglobin; HbA1C, glycosylated hemoglobin, type A1C; Hcy; homocysteine; L, lymphocytes; Lp‐PLA2, lipoprotein‐associated phospholipase A2; N, neutrophils; NLR, neutrophil lymphocyte ratio; PLT, platelets; SBP, systolic blood pressure; TNF‐α; tumor necrosis factor‐α; WBC, white blood cells.

### 3.5 Analysis of risk factors for carotid artery unstable plaque in ischemic stroke patients

A logistic regression model was established with the values of HBA1C, Lp‐pla2, CRP, CysC, Hcy, TNF‐α, N, NLR, Lp(a), TG, and TG/HDL‐C as independent variables and the nature of patients’ carotid plaques as dependent variables. The results showed that the increase in Lp(a), TG/HDL‐C, HBA1C, Lp‐PLA2, CRP, CysC, Hcy, and NLR could increase the risk of carotid artery unstable plaque in ischemic stroke patients (*p* < .05; Table 4).

**TABLE 4:** Analysis of risk factors of carotid unstable plaque in patients with ischemic stroke.

Index	*β*	SE	Walds	*p*	OR	95% CI	
Lp(a)	0.551	0.246	5.017	.034	1.735	1.071	2.810
TG	0.395	0.312	1.603	.264	1.484	0.805	2.736
TG/HDL‐C	0.601	0.274	4.811	.038	1.824	1.066	3.121
HBA1C	0.448	0.221	4.109	.048	1.565	1.015	2.414
Lp‐PLA2	0.574	0.250	5.272	.029	1.775	1.088	2.898
CRP	0.611	0.264	5.356	.027	1.842	1.098	3.091
CysC	0.57	0.241	5.594	.015	1.768	1.103	2.836
Hcy	0.475	0.230	4.265	.047	1.608	1.024	2.524
TNF‐α	0.533	0.301	3.136	.096	1.704	0.945	3.074
N	0.295	0.227	1.689	.251	1.343	0.861	2.096
NLR	0.601	0.246	5.969	.001	1.824	1.126	2.954
Constant term	1.044	0.507	4.240	.047	2.841	1.052	7.673

Abbreviations: CI, confidence interval; CRP, C‐reactive protein; CysC, photoprotein C; HbA1C, glycosylated hemoglobin, type A1C; Hcy; homocysteine; Lp(a), lipoprotein(a); N, neutrophils; NLR, neutrophil lymphocyte ratio; OR, odds ratio; Lp‐PLA2, lipoprotein‐associated phospholipase A2; TC, total cholesterol; TG, triglycerides; TG/HDL‐C, triglyceride/high‐density lipoprotein cholesterol; TNF‐α; tumor necrosis factor‐α.

## 4 DISCUSSION

Atherosclerosis is an important cause of ischemic stroke. Impaired lipid metabolism triggers atherosclerosis, and the accumulation of lipids and sugars in the intima or intimal bleeding and thrombus formation can lead to fibrous tissue proliferation as well as calcium accumulation, resulting in thickening of the arterial wall and narrowing of the arterial lumen. An increasing number of studies have shown that carotid unstable plaque detachment or rupture is more likely to cause ischemic stroke than arterial lumen narrowing (Baron et al., 2014; Ichinose et al., 2018; Skagen et al., 2016). Therefore, the control of risk factors for carotid unstable plaque dislodgement or rupture should be strengthened clinically. Therefore, it is important to explore the risk factors for carotid unstable plaque dislodgement or rupture for the prevention and treatment of ischemic stroke in the clinical setting.

In order to investigate the relationship between Lp(a), TG/HDL‐C, and carotid atherosclerotic plaque stability in ischemic stroke patients, this study first compared the general data of the two groups and found that there were no significant differences in age, BMI, gender, smoking, and alcohol consumption between the two groups, indicating that these factors were not risk factors for atherosclerotic plaque instability.

Lp(a) is a competitive inhibitor that inhibits fibrinolytic enzymes and can bind to vascular endothelial cells to promote thrombosis; in addition, Lp(a) inhibits fibrinogen activity and thrombolysis, thus causing disruption in the fibrinolytic system and promoting the formation of atherosclerosis (Bucci et al., 2016; Mathieu et al., 2017). HDL‐C can induce reverse cholesterol transport and thus exerts its anti‐atherogenic function, and reduced HDL‐C levels increase the instability of carotid plaques (W. Li, Liu, et al., 2021; Langlois et al., 2014). Related studies have reported that high levels of TG may cause some structural alterations in HDL‐C and LDL, and such alterations can be instrumental in promoting the progression of the lipid core in atherosclerotic plaques. The specific mechanism is that under the action of cholesterol transfer protein, TG in lipoprotein CM and VLDL exchanges lipid with cholesterol in lipoprotein LDL and HDL. The higher the level of TG, the more active the exchange, which leads to an increase in small and compact LDL levels, a decrease in the HDL level, an increase in TG‐rich lipoprotein residue level and cholesterol content, and finally an increase in atherogenic factors (Manjunath et al., 2013). It has also been reported in the literature that TG is an important risk factor for carotid plaque instability, with each 1 mmol/L increase in TG associated with a 1.07‐fold increase in the risk of unstable plaque formation, but the relationship between TG/HDL‐C and carotid plaque instability has not been fully explored (Lin et al., 2022). The main pathological feature of unstable plaques is a large lipid core, and oxidized LDL is a key influential molecule in carotid plaque instability (Mughal et al., 2011). Related studies have shown that TG/HDL‐C can effectively reflect LDL molecular particle size, and the TG/HDL‐C ratio is negatively correlated with LDL particle size, that is, the higher the TG/HDL‐C ratio, the smaller the LDL particles, and the easier the LDL particles are to be oxidized, thus increasing the incidence of atherosclerosis (Yokoyama et al., 2019). It was found that high levels of TG increase the risk of HDL lipolysis by hepatic lipase and that the inhibition of lecithin‐cholesterol acyltransferase and lipoprotein activity can affect HDL maturation (Tian & Fu, 2010). As the TG/HDL‐C ratio increases, the smaller LDL particles become more numerous and less resistant to atherosclerosis, and the larger lipid cores become more numerous. This will impede blood perfusion and the transport of nutrients in the blood, leading to the formation of an ischemic and hypoxic state in the carotid lumen, which slowly kills the outermost smooth muscle cells of the plaque. As a result, the fibrous cap becomes thinner, and plaque rupture is accelerated, and the ischemic stroke increases the risk of morbidity (Helkin et al., 2016). Therefore, TG/HDL‐C is an important influencing factor of atherosclerosis instability, but few studies have reported the relationship between TG/HDL‐C and carotid plaque instability. In this study, we further compared the lipid metabolism indexes of patients in groups A and B. The results showed that there were no significant differences in serum TC, HDL‐C, and LDL‐C between groups A and B, while the measured values of Lp(a), TG, and TG/HDL‐C in patients in group B were higher than those in group A. The results of this study indicated that Lp(a), TG, and TG/HDL‐C are important factors affecting the stability of atherosclerotic plaques, which is consistent with the results reported in the above related studies.

This study also investigated the diagnostic value of lipid metabolic indexes in unstable carotid atherosclerotic plaques in patients with ischemic stroke and found that Lp(a), TG, and TG/HDL‐C all have some diagnostic value for carotid atherosclerotic plaques as unstable plaques in ischemic stroke patients. The results indicate that these factors could be used to assess the stability of carotid plaques and should be given more clinical attention.

In this study, the comparison of routine blood, blood glucose, blood pressure, and inflammatory indexes between groups A and B revealed that the values of HBA1C, Lp‐PLA2, CRP, CysC, Hcy, TNF‐α, N, and NLR were higher in group B than in group A. These factors have been found to be the risk of unstable carotid plaque in patients with ischemic stroke. HBA1C can reflect patients’ blood glucose and is closely related to carotid intima‐media thickness, and high blood glucose can trigger vascular damage and abnormal endothelial cell function, so patients with high HBA1C are more likely to develop cardiovascular disease (Perlman et al., 2021; Schnell et al., 2017). Lp‐PLA2 can promote the production and accumulation of free fatty acids, which can contribute to the production and rupture of atherosclerotic plaques, thus triggering secondary thrombosis (Bonnefont‐Rousselot, 2016; X. Li, Xu, et al., 2021). CRP is one of the common clinical indicators of inflammation, and its binding to lipoproteins activates the production of substances related to vascular endothelial damage, which causes damage to the endothelium and leads to increased atherosclerotic plaque instability (Deme & Telekes, 2017). Related studies have reported that CysC overexpression increases vascular wall proteases, causing endothelial damage and leading to increased atherosclerotic plaque instability (Sarlak et al., 2014; Wu et al., 2014). Hcy has an inhibitory effect on the expression of nitric oxide synthase, which inhibits the production of the vasodilatory factor nitric oxide, promotes the synthesis of thromboxane and platelet aggregation, aggravates the damage to the vascular endothelium, and increases plaque instability (Bakoyiannis et al., 2015). NLRs have a catalytic effect on neutrophil activation within the plaque, leading to an accelerated progression of lesions in the vessel wall, resulting in the formation of non‐calcified plaques and mixed plaques, both of which are prone to rupture and dislodgement (R. Zhang et al., 2019). The inflammatory response plays an important role in the process of atherosclerosis, and the risk of plaque rupture is increased by accelerated macrophage infiltration in the presence of arterial injury, while TNF‐α is an important cytokine in the inflammatory response, so TNF‐α plays an important role in the development of atherosclerosis (Sengeløv et al., 2018; T. Wang & He, 2020). The results of this study further illustrate the important role of the above related indicators in the formation of atherosclerotic unstable plaques.

In conclusion, Lp(a) and TG/HDL‐C are valuable in assessing carotid atherosclerotic plaque stability in patients with acute ischemic stroke, and their elevated levels significantly increase the risk of developing unstable carotid plaques in patients.

## AUTHOR CONTRIBUTIONS

Yanan Tian and Jianhui Wei designed the study. Yanan Tian and Lei Lu performed the study. Yazhao Zhang analyzed the data. All authors have participated sifficiently in the work and agreed to be accountable for all aspects of the work. All authors read and approved the final manuscript.

## CONFLICT OF INTEREST STATEMENT

The authors declare no conflicts of interest.

## FUNDING INFORMATION

No funding was received for this research.

### PEER REVIEW

The peer review history for this article is available at https://publons.com/publon/10.1002/brb3.3355.

## Data Availability

The datasets generated or analyzed during this study are available from the corresponding author upon reasonable request.
